# Case Report: Anti-MOG Antibody Seroconversion Accompanied by Dimethyl Fumarate Treatment

**DOI:** 10.3389/fimmu.2021.625465

**Published:** 2021-02-15

**Authors:** Keita Takahashi, Hideyuki Takeuchi, Ryoko Fukai, Haruko Nakamura, Keisuke Morihara, Yuichi Higashiyama, Toshiyuki Takahashi, Hiroshi Doi, Fumiaki Tanaka

**Affiliations:** ^1^Department of Neurology and Stroke Medicine, Yokohama City University Graduate School of Medicine, Yokohama, Japan; ^2^Department of Neurology, Tohoku University Graduate School of Medicine, Sendai, Japan

**Keywords:** anti-myelin oligodendrocyte glycoprotein antibody-associated disease, dimethyl fumarate, fingolimod, multiple sclerosis, seroconversion

## Abstract

Here we report three cases of anti-myelin oligodendrocyte glycoprotein (MOG) antibody–associated disease (MOGAD) mimicking multiple sclerosis in which seropositivity for anti-MOG antibodies occurred during disease-modifying drug dimethyl fumarate (DMF) treatment. These patients developed relapses with anti-MOG antibody seroconversion after switching from fingolimod or steroid pulse therapy to DMF, which was associated with peripheral lymphocyte recovery. MOGAD is considered a humoral immune disease, and DMF reportedly enhances Th2-skewed humoral immune activity. Therefore, we suggest that DMF, but not fingolimod, may exacerbate humoral immune imbalance and enhance autoantibody production, leading to aggravation of MOGAD.

## Introduction

Anti-myelin oligodendrocyte glycoprotein (MOG) antibody–associated disease (MOGAD), which makes up ~40% of anti-aquaporin 4 (AQP4) antibody–negative neuromyelitis optica spectrum disorders, is often difficult to distinguish from multiple sclerosis (MS) (1). Whether disease-modifying drugs (DMDs) widely used in MS are also effective in MOGAD remains unclear. Here we report three cases of MOGAD treated as atypical MS due to negativity for specific antibodies in which seroconversion for anti-MOG antibodies occurred when DMD was switched to dimethyl fumarate (DMF).

## Case Report

**Figure 1:**
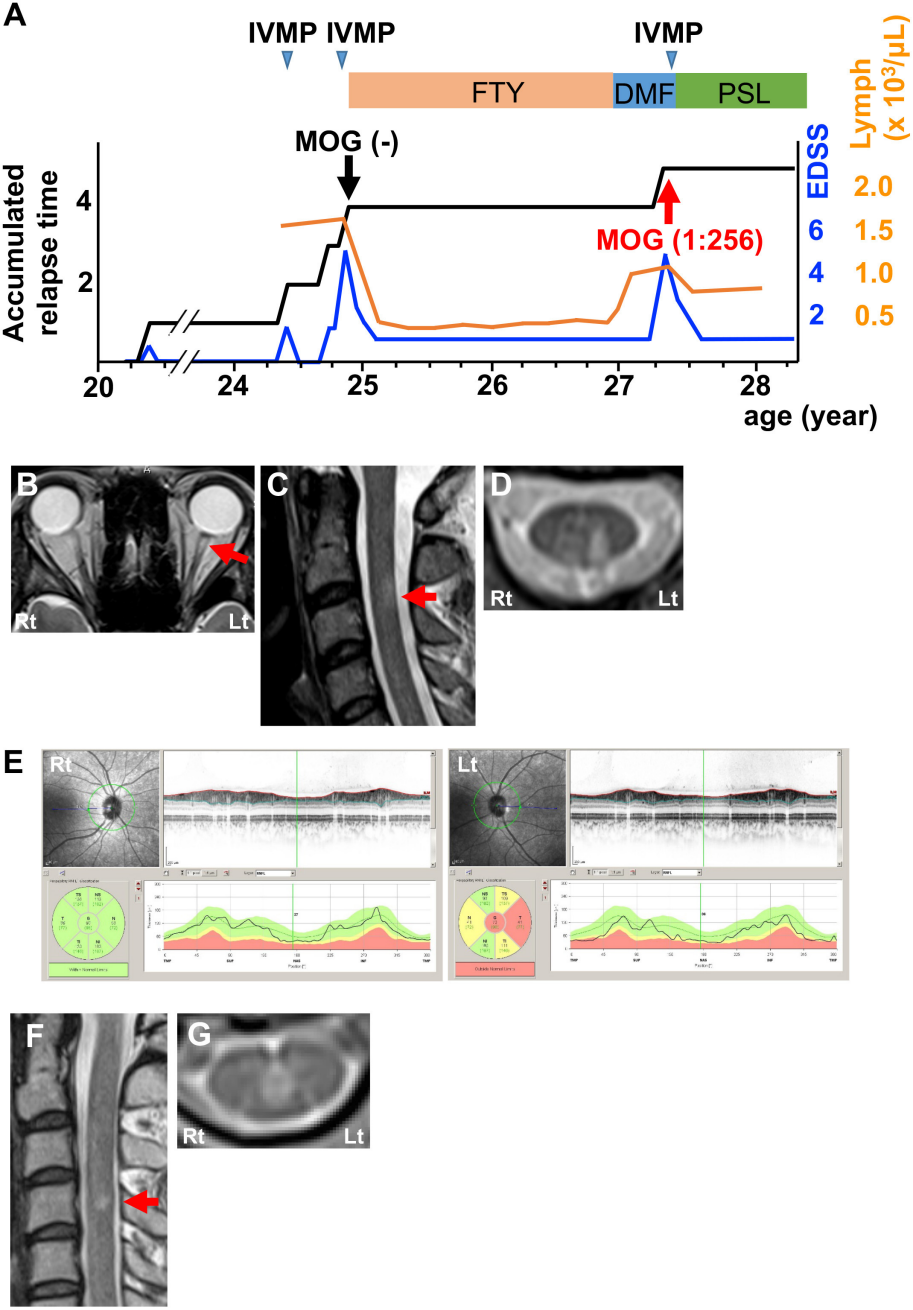
Clinical features of Patient 1. **(A)** Graph of the time course for relapses and treatments. **(B)** Axial T2-weighted orbit MRI showing left optic nerve lesion (red arrow). **(C)** Sagittal T2-weighted cervical MRI showing a short cervical lesion at the C3 level (red arrow). **(D)** Axial T2-weighted cervical MRI showing a lesion in the left dorsal horn and fasciculus at the C3 level. **(E)** OCT showing significant RNFL thinning in the left eye. Green, normal RNFL thickness (5th−95th percentile); yellow, mild-to-moderate RNFL thinning (1st−5th percentile); red, severe RNFL thinning (<1st percentile). **(F)** Sagittal T2-weighted cervical MRI showing a short cervical lesion at the C4 level (red arrow). **(G)** Axial T2-weighted cervical MRI showing a lesion around the central canal at the C4 level. DMF, dimethyl fumarate; FTY, fingolimod; IVMP, intravenous methylprednisolone pulse therapy; PSL, prednisolone; EDSS, Expanded Disability Status Scale score; Lymph, peripheral lymphocyte count.

The first patient was a 27-year-old woman who experienced two attacks of optic neuritis and transverse myelitis with short cervical and thoracic cord lesions in 4 years (Figure 1A). She experienced severe attacks of left retrobulbar neuritis and transverse myelitis at the age of 24. Magnetic resonance imaging (MRI) showed T2-hyperintense lesions in the left optic nerve (Figure 1B) and left dorsal horn and fasciculus at the C3 level (Figures 1C,D). Optical coherence tomography (OCT) detected a significant decrease in retinal nerve fiber layer (RNFL) thickness in the left eye (Figure 1E). Cerebrospinal fluid (CSF) analysis revealed a slight elevation in protein levels and increases in myelin basic protein (MBP) levels and IgG index, but not in cell count or oligoclonal bands (Table 1; Patient 1, pre). Neither anti-AQP4 nor anti-MOG antibodies were detected in serum samples using an internationally standardized cell-based assay (CBA) (2–4). She had been effectively treated with fingolimod for the diagnosis of atypical MS for 2.5 years. Since she desired to have a child, fingolimod was switched to DMF. Three months later, she developed a severe relapse of transverse myelitis with a short cervical cord lesion accompanied by recovery from lymphocytopenia (Figure 1A). MRI showed T2-hyperintense lesion around the central canal at the C4 level (Figures 1F,G). CSF analysis also indicated elevation of protein levels, MBP levels, and IgG index, but pleocytosis and oligoclonal bands were not detected (Table 1; Patient 1, post). At that time, the second CBA using the same methodology (3) revealed seroconversion for anti-MOG antibodies (titer, 1:256; cut-off, ≥1:128). We reconfirmed that the frozen serum sample from the time of the first CBA was truly seronegative for anti-MOG antibodies. Intravenous methylprednisolone pulse therapy (1 g/day for 3 consecutive days; IVMP) markedly decreased symptoms. She has had no relapses with subsequent oral prednisolone therapy (20 mg/day) for over 1 year (Figure 1A).

**Table 1:** CSF findings of three patients.

	**Patient 1** **pre/post**	**Patient 2** **pre/post**	**Patient 3** **pre/post**
Cell count (cells/μl)	2/2	20/10	2/1
Protein (mg/dl)	44/86	51/31	31/31
Myelin basic protein (pg/ml)	86.5/103.1	ND/ND	1729.8/ND
IgG index	0.91/0.64	2.06/1.36	0.36/0.41
Oligoclonal band	–/–	+/+	–/–

*Pre, CSF collection before seroconversion for anti-MOG antibodies; post, CSF collection after seroconversion for anti-MOG antibodies; ND, not detected*.

**Figure 2:**
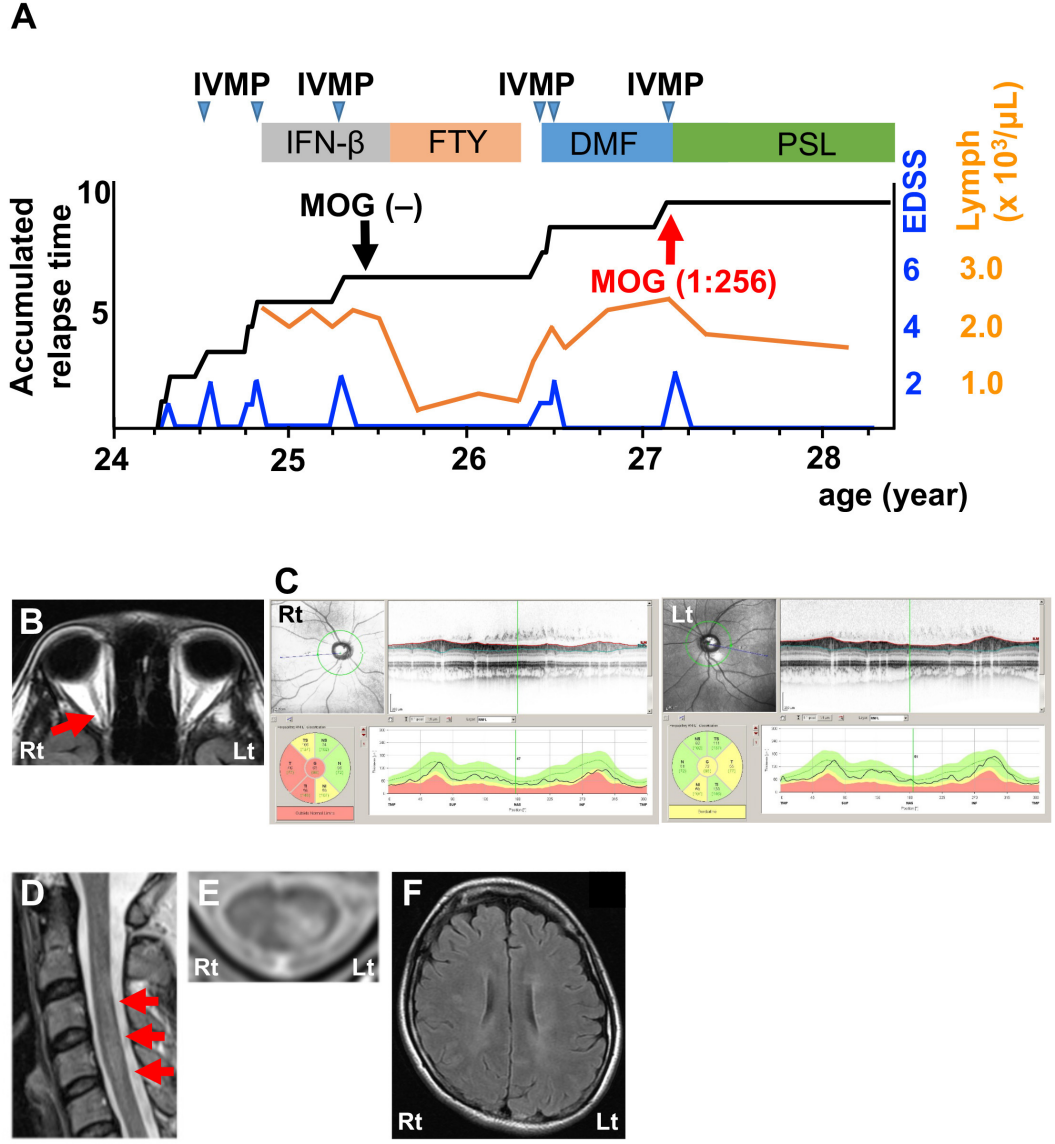
Clinical features of Patient 2. **(A)** Graph of the time course for relapses and treatments. **(B)** Axial FLAIR orbit MRI showing right optic nerve lesions (red arrow). **(C)** OCT showing severe RNFL thinning in the right eye and mild RNFL thinning in the left eye. Green, normal RNFL thickness (5th−95th percentile); yellow, mild-to-moderate RNFL thinning (1st−5th percentile); red, severe RNFL thinning (<1st percentile). **(D)** Sagittal T2-weighted cervical MRI showing a lesion at the C3–4 levels (red arrows). **(E)** Axial T2-weighted cervical MRI showing lesions in the left dorsal horn and fasciculus at the C4 level. **(F)** Axial FLAIR brain MRI showing periventricular and subcortical lesions. DMF, dimethyl fumarate; FTY, fingolimod; IFN-β, interferon-β; IVMP, intravenous methylprednisolone pulse therapy; PSL, prednisolone; EDSS, Expanded Disability Status Scale score; Lymph, peripheral lymphocyte count.

The second patient was a 27-year-old woman who had several attacks of optic neuritis and transverse myelitis involving short lesions in the cervical cord in a year (Figure 2A). She experienced a severe attack of optic neuritis around the age of 25, as shown on fluid-attenuated inversion recovery (FLAIR) MRI (Figure 2B). OCT revealed severe RNFL thinning in the right eye and mild RNFL thinning in the left eye (Figure 2C). CSF analysis detected pleocytosis, elevations in protein levels and IgG index, and the presence of oligoclonal bands, but not MBP (Table 1; Patient 2, pre). A CBA did not detect anti-AQP4 or anti-MOG antibodies in the serum. She had been treated with interferon-β followed by fingolimod for the diagnosis of atypical MS for 1 year without any relapses, but DMD was changed to DMF because of epidural hematoma as an unexpected adverse side effect of fingolimod (5). After 1 year, she had a relapse of transverse myelitis with a short cervical cord lesion (Figures 2D,E) and asymptomatic MS-like supratentorial lesions (Figure 2F) following lymphocyte recovery (Figure 2A). CSF analysis revealed a profile that was similar to the profile before DMF treatment, except for normal protein levels at this time (Table 1; Patient 2, post). This patient also had seroconversion for anti-MOG antibodies (titer, 1:256) using the same methodology. Serum from the time of the first CBA was reconfirmed to be truly seronegative. IVMP substantially decreased her symptoms. Oral prednisolone (20 mg/day) completely suppressed the relapse for more than a year (Figure 2A).

**Figure 3:**
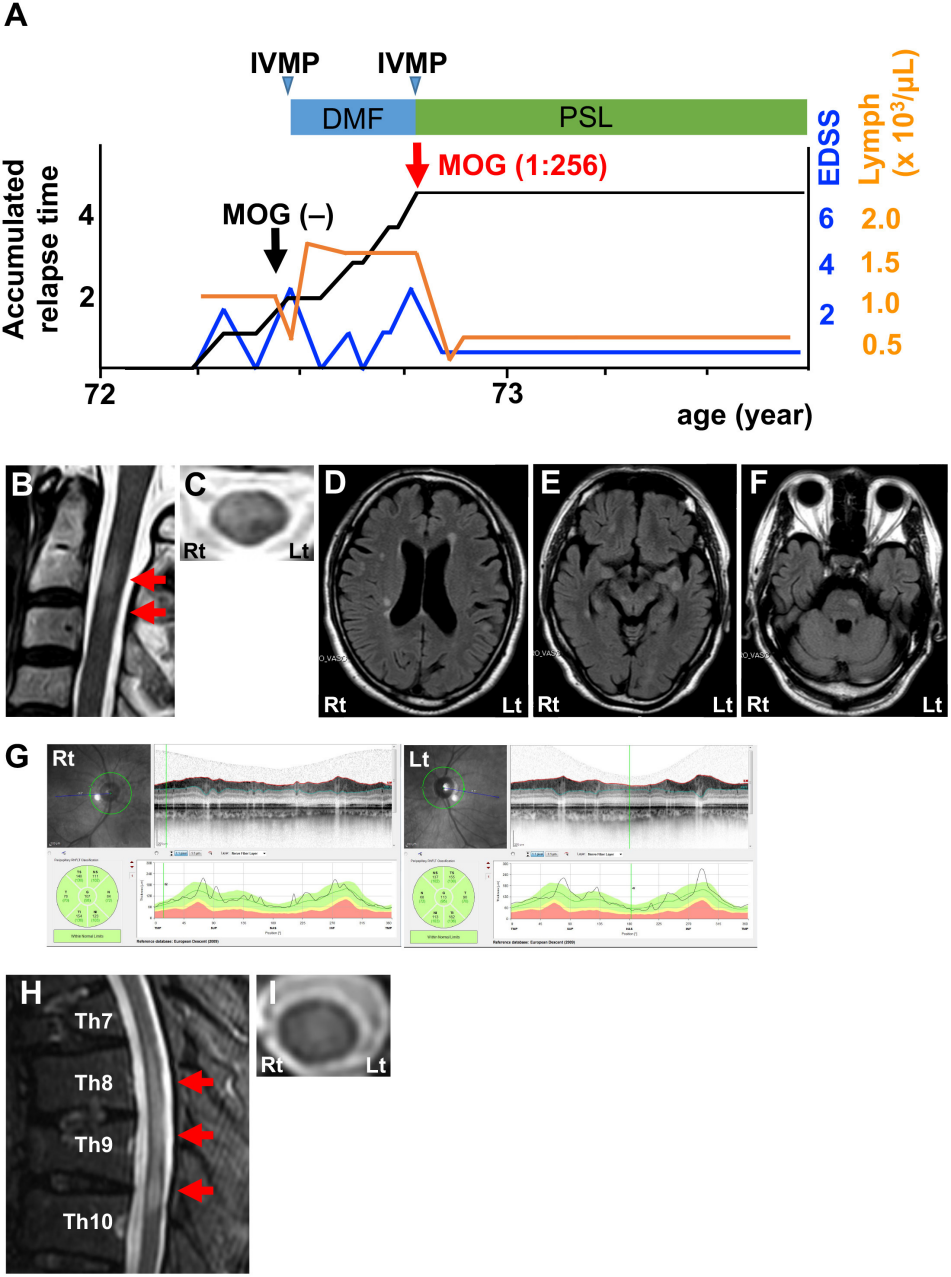
Clinical features of Patient 3. **(A)** Graph of the time course for relapses and treatments. **(B)** Sagittal T2-weighted cervical MRI showing short lesions at the C2–3 level (red arrows). **(C)** Axial T2-weighted cervical MRI showing a ventral-dominant lesion at the C2 level. **(D–F)** Axial FLAIR brain MRI showing lesions in the periventricular area, subcortical area, and brain stem. **(G)** OCT showing normal RNFL thickness. Green, normal RNFL thickness (5th−95th percentile). **(H)** Sagittal T2-weighted thoracic MRI showing lesions at the Th8–10 levels (red arrows). **(I)** Axial T2-weighted cervical MRI showing lesion at the Th8 level around the central canal. DMF, dimethyl fumarate; IVMP, intravenous methylprednisolone pulse therapy; PSL, prednisolone; EDSS, Expanded Disability Status Scale score; Lymph, peripheral lymphocyte count.

The third patient was a 73-year-old man with a severe attack of transverse myelitis in the previous year (Figure 3A). FLAIR MRI showed short lesions in the cervical cord (Figures 3B,C) as well as asymptomatic lesions in the periventricular area, subcortical area, and brain stem (Figures 3D–F). OCT indicated no signs of optic neuritis (Figure 3G). CSF analysis revealed a prominent increase in MBP levels, but was otherwise normal (Table 1; Patient 3, pre). Neither anti-AQP4 nor anti-MOG antibodies were detected in the serum using the same CBA described for the other 2 patients. IVMP effectively resolved his symptoms. He was subsequently treated with DMF for the diagnosis of atypical MS. Lymphocyte counts became higher than pre-treatment levels and reached a plateau (Figure 3A). Three months later, he developed a severe relapse of transverse myelitis. MRI revealed a FLAIR-hyperintense lesion around the central canal extending from the Th8 level to the Th10 level (Figures 3H,I). CSF analysis showed a normal profile, including normal MBP levels (Table 1; Patient 3, post). A CBA for anti-MOG antibodies turned positive at that time (titer, 1:256). We also reconfirmed seronegativity for anti-MOG antibodies in a frozen serum sample from the time of the first CBA. IVMP was effective in decreasing his symptoms again. To date, he has been in remission with oral prednisolone therapy (20 mg/day) for more than a year (Figure 3A).

## Discussion

Although therapy for MOGAD is yet to be established, according to consensus among international experts (6, 7), immunosuppressive therapy (e.g., corticosteroids, azathioprine, tacrolimus, mycophenolate mofetil, and methotrexate) is the mainstay for treatment to prevent relapse. In particular, B cell depleting therapies such as rituximab have shown good therapeutic responses, but relapse occurs immediately after B cell recovery. These data strongly suggest that suppression of humoral immunity is likely the key therapeutic strategy for MOGAD. DMDs for MS such as interferon-β, glatiramer acetate, and natalizumab might not be efficacious, but the effectiveness of fingolimod remains uncertain (6, 7). Although a recent report mentioned that DMF is ineffective but not harmful in a patient with MOGAD (8), the effectiveness of DMF has yet to be determined.

Initially, our patients were diagnosed with atypical MS because they were seronegative for specific antibodies and had good response to IVMP and fingolimod. However, seroconversion for anti-MOG antibodies occurred when DMD therapy was switched to DMF. We propose two possible mechanisms to explain this seroconversion. One possibility is that the first CBA results in all patients were false-negative due to fluctuations in autoantibody titers, and the timing of seroconversion and switching to DMF was just a coincidence because the titers from the second CBA in all patients were relatively low (1:256). Despite low titers of anti-MOG antibodies, all patients have been in remission with oral prednisolone therapy for at least 1 year after their last relapse. This good response to corticosteroid therapy suggests that these patients truly had MOGAD. Another possibility is that DMF treatment enhanced anti-MOG autoantibody production whereas fingolimod treatment effectively suppressed it. The main therapeutic effect of fingolimod is immunosuppression due to the retention of auto-reactive lymphocytes within secondary lymphoid organs, whereas the net activity of DMF is mainly a pro-tolerogenic lymphocyte shift (9). As humoral immunosuppression, fingolimod is likely efficacious in MOGAD whereas DMF is not efficacious. A previous report documented a similar seroconversion for anti-MOG antibodies during interferon-β treatment, which modulates immune balance from Th1/Th17-mediated cellular immunity to Th2-mediated humoral immunity and may promote autoantibody synthesis (10). Like interferon-β, DMF also enhances Th2-skewed humoral immune activity (11). Another report demonstrated that DMF induces severe relapses of neuromyelitis optica spectrum disorders, indicating that DMF has a detrimental effect in humoral immunity-mediated diseases (12). In fact, our cases revealed that fingolimod effectively suppresses peripheral lymphocyte count and switching from fingolimod to DMF or initiating DMF leads to a recovery of peripheral lymphocyte count accompanied by seroconversion for anti-MOG antibodies (Figures 1A, 2A, 3A). These findings suggest that DMF might exacerbate Th2-prone humoral autoimmunity. In addition, two of three patients (Patients 1 and 2) had similar or higher peripheral lymphocyte counts at the time of the first CBA than at the time of the second CBA, suggesting that the Th2-skewed peripheral lymphocyte subpopulation shift associated with DMF (11) may affect MOGAD development rather than the higher absolute number of peripheral lymphocytes.

In conclusion, we presented three cases of MOGAD with seroconversion for anti-MOG antibodies accompanied by recurrence of clinical disability after treatment with DMF. Thus, DMF should be avoided in MOGAD, whereas fingolimod may be efficacious because it suppresses humoral immunity.

## Practical Implications

Dimethyl fumarate may exacerbate anti-myelin oligodendrocyte glycoprotein antibody–associated disease whereas fingolimod may be effective against this disease.

## Data Availability Statement

The original contributions presented in the study are included in the article/supplementary material, further inquiries can be directed to the corresponding authors.

## Ethics Statement

Written informed consent was obtained from the patients for the publication of this case series in accordance with the Declaration of Helsinki.

## Author Contributions

KT, HT, RF, HN, KM, YH, and HD examined and treated the patient. TT assessed anti-MOG antibody titers. KT and HT analyzed data. HT and FT designed and supervised this study. KT, HT, and FT wrote the manuscript. All authors contributed to the article and approved the submitted version.

## Conflict of Interest

The authors declare that the research was conducted in the absence of any commercial or financial relationships that could be construed as a potential conflict of interest.
